# Validation of urine colour L*a*b* for assessing hydration amongst athletes

**DOI:** 10.3389/fnut.2022.997189

**Published:** 2022-08-10

**Authors:** Yiwei Feng, Guoliang Fang, Chaoyi Qu, Shuqiang Cui, Xue Geng, Derun Gao, Fei Qin, Jiexiu Zhao

**Affiliations:** ^1^Exercise Biological Center, China Institute of Sport Science, Beijing, China; ^2^Department of Exercise Physiology, Beijing Sport University, Beijing, China; ^3^Beijing Institute of Sports Science, Beijing, China; ^4^Institute of Sports Medicine and Health, Chengdu Sport University, Chengdu, China; ^5^School of Physical Education, Jinan University, Guangzhou, China

**Keywords:** hydration, validity, athlete, urine colour, colour space, CIE L*a*b*

## Abstract

**Objectives:**

Existing studies have confirmed that urine colour through a urine colour chart is one of the effective indicators for assessing hydration. In recent years, the L*a*b* colour space has been widely used in the objective quantitative analysis of colour. The L*, a* and b* values represent the luminance change from black to white, the chromaticity change from green to red and the chromaticity change from blue to yellow, respectively. This study aimed to examine the validity of the urine colour L*a*b* parameters for assessing the level of hydration amongst athletes.

**Methods:**

The study included a total of 474 young elite athletes (251 males and 223 females, age: 24.59 ± 4.86 years). A total of 803 urine samples were collected from the subjects in various stages of hydration, including morning urine and spot urine sample during rehydration. L*a*b* parameters were measured by spectrophotometer. Hydration status was assessed via urine osmolality and urine specific gravity.

**Results:**

Urine colour b* value has a high correlation with urine specific gravity and urine osmolality (r = 0.811, 0.741, both *p* < 0.01); L* value has a moderate correlation with urine specific gravity and urine osmolality (r = –0.508, –0.471, both *p* < 0.01); there was no significant correlation between a* value and urine specific gravity, urine osmolality (*p* > 0.05). Whether the diagnosis of hypohydration is based on Usg ≥ 1.020 or Uosm ≥ 700 mmol/kg: The AUC of b* values were all above 0.9 and the specificity and sensitivity of b* values were high (both greater than 80%). The AUC of both L* and a* values were less than 0.5. Whether the diagnosis of hyperhydration is based on Usg ≤ 1.010 or Uosm ≤ 500 mmol/kg: The AUC of b* values were all above 0.9 and the specificity and sensitivity of b* value were high (both greater than 90%). The AUC of both L* and a* values were less than 0.5.

**Conclusion:**

These results suggested that the validity of urine colour b* value for assessing hydration amongst athletes was high, however, the validity of urine colour L* and a* values were low.

## Introduction

Maintaining proper hydration is essential for athletes to maintain optimal performance and health (1). A level of hypohydration (greater than 1% of body weight) can impair thermoregulation and cognitive function. Hypohydration (> 2% of body weight) can degrade endurance performance (2). There are many methods for assessing hydration status. Urine testing is a common non-invasive method for monitoring hydration and is highly accepted by athletes. Urine colour, urine specific gravity (Usg) and urine osmolality (Uosm) are valid indices of hydration status (3, 4).

Urine colour can be measured visually using the urine colour chart. Armstrong et al. (3, 5) first developed the urine colour chart and then proved that urine colour is an effective index in assessing hydration through a urine colour chart. However, urine colour charts are easily affected by factors such as test environment, container material, urine volume and operator’s subjective perception. Given the age differences, renal urine concentration and excretion function amongst different populations, the validity of the urine colour chart has also been questioned. The correlation coefficient r between urine colour and urine specific gravity or urine osmolality is between 0.40–0.93. Urine colour charts are not equally effective in assessing hydration of different populations such as general adults (6), young athletes (3, 5), nursing home residents (7), children (8), pregnant and nursing women (9).

Colour is a subjective feeling. Researchers developed colour space models to quantitatively and objectively analyse colour. Colour space models record colours in the form of abstract mathematical models. There are various colour space modes, commonly used are RGB, CMYK, L*a*b*, HSV and so on. The L*a*b* colour space model was developed by the International Commission on Illumination (CIE) in 1976 and includes all colours visible to the human eye. The L*a*b* colour model has a larger colour gamut range than the RGB and CMYK colour models. The L*, a* and b* values represent the change in brightness from black (0) to white (100), from green (−128) to red (+ 127) and from blue (−128) to yellow (+ 127) (10), respectively. Therefore, urine colour can be quantitatively represented by the L*a*b*colour space.

In addition to urine colour charts, urine colour can be measured using instruments. A spectrophotometer is a common instrument for measuring the colour of solids and liquids. It is easy to operate, fast to measure and is not affected by the operator’s subjective perception and external environment. In addition, spectrophotometer has been used in food, agriculture, printing, construction, chemical, pharmaceutical, textile and clothing, coatings elite and other industries. It can convert optical signals into electrical signals and digital signals, thus, quantitatively analyse the colour parameters.

In recent years, researchers have tried to quantitatively analyse urine colour L*a*b* through the use of instruments (11–14). Renal function varies amongst populations, hence, the validity of urine colour L*a*b* parameters for assessing hydration of athletes is unclear. Currently, no studies have been reported on urine colour L*a*b* amongst athletes. We are not yet sure which L*a*b* parameter is the most effective hydration indicator for assessing athletes. This study aimed to study the correlations between urine colour L*a*b* and urine concentration, investigate the diagnostic ability of urine colour L*a*b* for hypohydration and hyperhydration as defined by urine concentration and identify the threshold value and examine the validity of the urine colour L*a*b* for assessing hydration amongst young and healthy athletes.

## Methods and materials

### Participants

The subjects of this study were all elite athletes from China. Sports events included basketball, boxing, diving, track and field, kayaking, track cycling, swimming, trampoline, gymnastics, shooting, synchronised swimming, weightlifting, rock climbing, judo, beach volleyball, triathlon and so on.

Inclusion criteria: Active athletes who are highly-trained and healthy in status.

Exclusion criteria: athletes that are suffering from endocrine diseases, urinary system diseases, diabetes, heart disease and other kinds of diseases; taking drugs including diuretics within one week; and women with recent menstrual bleeding.

### Study procedure

We experimented in two phases to collect a large sample size of urine samples from athletes in different stages of hydration.

Procedure 1: A cross-sectional study was conducted on a total of 529 morning urine samples that were collected from 419 athletes of different sports. We repeatedly collected urine samples from some athletes during different training cycles. Subjects were instructed to refrain from consuming caffeine and alcohol for 24 h before the test. Urine samples were taken after getting up in the morning, before eating and rehydrating.

Procedure 2: We conducted a water-drinking intervention study amongst 55 athletes. Subjects were instructed to fast for 1 h and refrain from taking caffeine and alcohol for 24 h before the test. In the experiment, participants were encouraged to drink 100–200 mL of pure water every 10–20 minutes. Urine samples should be taken when the subjects feel urinating during rehydration, and urine specific gravity was tested immediately by the tester. When urine specific gravity of the urine sample was less than 1.005, the subject ended drinking. During this procedure, a total of 274 urine samples were collected.

In all, we collected a total of 803 urine samples through two procedures, which were subsequently tested and analysed together.

### Urine analysis

After the urine samples were collected, the test was immediately completed. If the urine sample cannot be tested in time, it should be stored in a refrigerator at 5 ± 1°C and tested within 7 days (15, 16). Urine osmolality was measured in duplicate using a Vapor Osmometer (Vapro 5600, Wescor, United States). Urine specific gravity was measured in duplicate using a digital refractometer (PAL-10S, Atago, Tokyo, Japan). If the difference between the first two measurements was greater than 0.0005, the tester took the third measurement and chose the median (16). Urine colour L*a*b* parameters were measured using a benchtop spectrophotometer (CR-5, Konica Minolta, Japan) with a 10mm pathlength quartz cuvette. The urine colour distribution map and L*a*b* data were recorded using Spectra Magic NX software (Konica Minolta Photo Imaging LTD., HK) (13).

### Urinary definition of hydration

Based on expert opinion and scientific research (17–21), hypohydration was defined as urine specific gravity of ≥1.020 and urine osmolality of ≥700 mmol/kg. Euhydration was defined as urine specific gravity in the range of 1.010–1.020 and urine osmolality in the range of 500–700 mmol/kg. Meanwhile, hyperhydration was defined as urine specific gravity of ≤1.010 and urine osmolality ≤ 500 mmol/kg.

### Statistical analysis

Data were analysed using IBM SPSS Statistics version 26.0. The quantitative parameters of participants were expressed as Mean ± SD. Relationships between parameters were investigated by Pearson correlation analysis and regression analysis. The level of statistical significance was set as *p* < 0.05. Receiver operating characteristics (ROC) curves, the area under the curve (AUC), sensitivity and specificity were calculated to determine urine colour L*a*b* parameters’ diagnostic ability to assess hydration based on the correct classified urine samples for urine concentration. The optimal threshold value of urine colour L*a*b* parameters for assessing hydration was determined using the Youden index.

## Results

### Participants and urine sample characteristics

We collected a total of 803 urine samples from 474 athletes through the cross-sectional study and the water-drinking intervention study. Means and standard deviations of all participants and urine sample characteristics are presented in Table 1.

**TABLE 1:** Participants and urine sample characteristics.

Variable	Mean ± SD	Range
Subjects (male/female)	474(251/223)	474(251/223)
Age (years)	24.59 ± 4.86	15–38
Height (cm)	178.64 ± 9.40	152.00–225.00
Weight (kg)	72.52 ± 13.51	36.00–149.00
Urine specific gravity	1.022 ± 0.008	1.0013–1.0381
Urine osmolality (mmol/kg)	780.69 ± 272.97	49.00–1260.00
L* (units)	93.34 ± 6.39	50.62–100.97
a* (units)	1.18 ± 1.92	−5.22–11.41
b* (units)	26.45 ± 11. 60	1.38–63.14

### Urine colour distribution map

**FIGURE 1:**
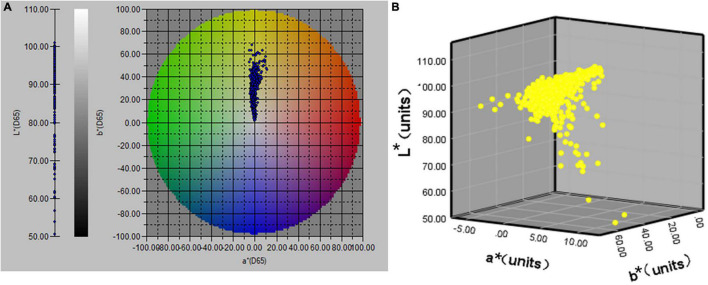
The distribution map of CIE L*a*b* values of urine samples (n = 803); **(A)** distribution of L*a*b* values in a 2D space; **(B)** distribution of L*a*b* values in a 3D space.

Urine colour can be objectively visualised in the CIE L*a*b* colour space by extracting the colour data and pictures in the Spectra Magic NX software. The urine colour distribution map is shown in Figure 1.

### Hydration status

When hydration was defined as urine specific gravity, a total of 560, 85 and 158 urine samples indicated dehydration, hyperhydration and euhydration, which accounted for a proportion of 69.7, 10.6 and 19.7%, respectively.

When hydration was defined as urine osmolality, a total of 564, 132 and 107 urine samples indicated dehydration, hyperhydration and euhydration, which accounted for a proportion of 70.2, 16.4 and 13.3%, respectively.

### Relationship between urine specific gravity and urine osmolality

**FIGURE 2:**
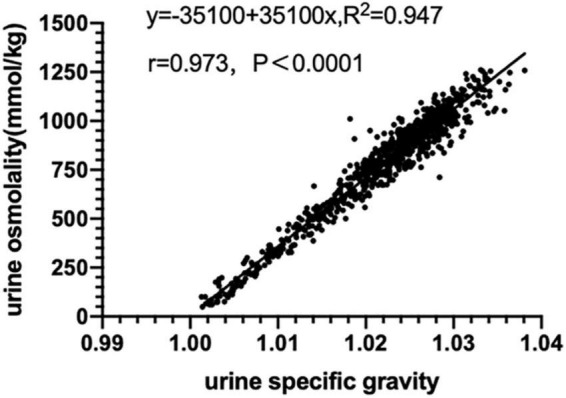
Relationship between urine specific gravity and urine osmolality (*n* = 803).

There was a high correlation between urine specific gravity and urine osmolality (*r* = 0.973, *p* < 0.01) and there was a linear regression equation (Uosm = −35100 + 35100 Usg, R^2^ = 0.947) as shown in Figure 2.

### Relationship between urine colour L*a*b* and urine concentration

There was a high positive correlation found between urine osmolality, urine specific gravity and urine colour b* value (*r* = 0.741, 0.811, both *p* < 0.01), and there was a linear regression equation. These results indicated that as dehydration increased, urine colour gradually turned yellow, the b* value gradually increased along the blue-yellow axis, showing a linear trend. Overall, the b* value had high validity in assessing hydration amongst athletes.

There was a moderate negative correlation found between urine osmolality, urine specific gravity and L* value (*r* = −0.471, −0.508, both *p* < 0.01), and there was a linear regression equation. These results indicated that as dehydration increased, urine colour gradually turned dark and the L* value gradually decreased along the white-black axis. Overall, the L* value had moderate validity in assessing the hydration of athletes.

**FIGURE 3:**
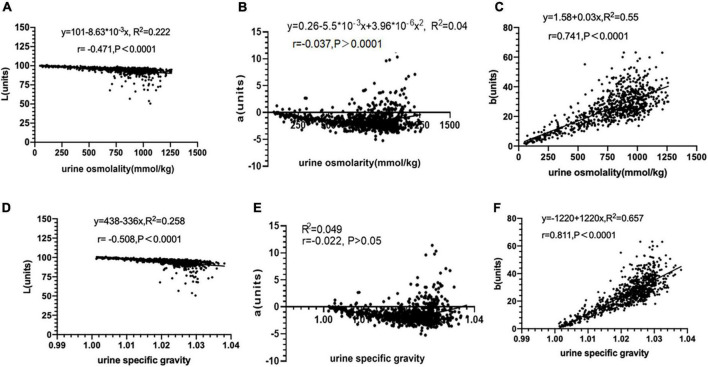
Relationship between urine colour L*a*b* and urine concentration (*n* = 803). **(A)** The relationship between urine color L* value and urine osmolality. **(B)** The relationship between urine color a* value and urine osmolality. **(C)** The relationship between urine color b* value and urine osmolality. **(D)** The relationship between urine color L* value and urine specific gravity. **(E)** The relationship between urine color a* value and urine specific gravity. **(F)** The relationship between urine color b* value and urine specific gravity.

There was no significant correlation found between urine osmolality, urine specific gravity and a* value (*p* > 0.05). The fitting curve was almost parabolic, the a* value first decreased and then increased along the green-red axis, roughly showing a parabolic trend. However, the regression equation was not significant, suggesting the a* value is not feasible to assess hydration amongst athletes (Figure 3). The above results were roughly in line with the actual situation. As the degree of dehydration increased, the urine colour gradually became darker, the brightness gradually decreased, and the chroma gradually became yellow.

### Validity of urine colour L*a*b* for assessing hypohydration

**FIGURE 4:**
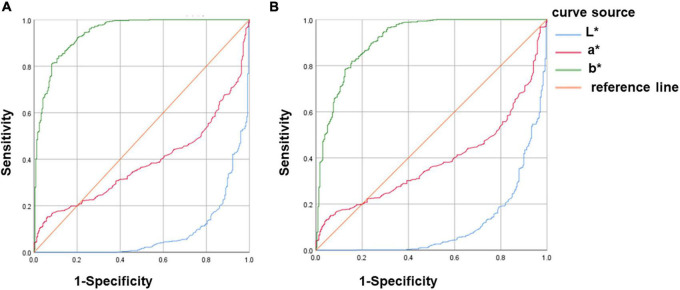
ROC curve analysis of urine colour L*a*b* for assessing hypohydration. **(A)** Hypohydration was defined as Usg ≥ 1.020. **(B)** Hypohydration was defined as Uosm ≥ 700mmol/kg; Uosm, urine osmolality; and Usg, urine specific gravity.

Whether the diagnosis of hypohydration is based on Usg ≥ 1.020 or Uosm ≥ 700 mmol/kg: The AUC of b* values were all above 0.9, the specificity and sensitivity of b* values were high (both greater than 80%), and the threshold value of b* for diagnosing hypohydration was 22.61. This suggests that the b* value had high validity in assessing hypohydration amongst young athletes. The AUC of both L* and a* values were less than 0.5, suggesting that L* and a* values had no diagnostic effect on hypohydration (Figure 4 and Table 2).

**TABLE 2:** ROC curve analysis of urine colour L*a*b* to identify hypohydration.

Predictive variable	Diagnostic standard	Threshold	AUC	Sensitivity(%)	Specificity(%)
L*	Uosm	49.62	0.109	100	0
a*	Uosm	0.045	0.388	15.1	6.3
b*	Uosm	22.61	0.914	81.9	84.5
L*	Usg	49.62	0.085	100	0
a*	Usg	0.045	0.385	15.2	93.9
b*	Usg	22.61	0.942	84.4	88.6

The predictive variable was tested against the corresponding hypohydration diagnostic standard Usg ≥ 1.020, Uosm ≥ 700 mmol/kg from the urine samples; Uosm, urine osmolality; Usg, urine specific gravity.

### Validity of urine colour L*a*b* for assessing hyperhydration

**FIGURE 5:**
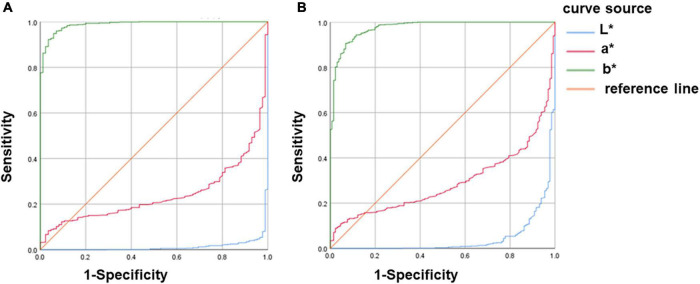
ROC curve analysis of urine colour L*a*b* for assessing hyperhydration. **(A)** Hyperhydration was defined as Usg ≤ 1.010. **(B)** Hyperhydration was defined as Uosm ≤ 500mmol/kg; Uosm, urine osmolality; and Usg, urine specific gravity.

Whether the diagnosis of hyperhydration was based on Usg ≤1.010 or Uosm ≤500 mmol/kg: The AUC of b* values were all above 0.9 and the specificity and sensitivity of b* values were high (both greater than 90%), which suggests that the b* value has high validity in assessing hyperhydration amongst young athletes. The AUC of both L* and a* values were less than 0.5, suggesting that L* and a* values had no diagnostic effect on hyperhydration (Figure 5 and Table 3).

**TABLE 3:** ROC curve analysis of urine colour L*a*b* to identify hyperhydration.

Predictive variable	Diagnostic standard	Threshold	AUC	Sensitivity(%)	Specificity(%)
L*	Uosm	49.62	0.043	100	0
a*	Uosm	0.725	0.291	8.9	97.7
b*	Uosm	17.395	0.975	90.6	93.1
L*	Usg	49.62	0.013	100	0
a*	Usg	0.725	0.241	8.4	96.5
b*	Usg	13.48	0.989	94.8	94.1

The predictive variable was tested against the corresponding hypohydration diagnostic standard Usg ≤ 1.010, Uosm ≤ 500 mmol/kg from the urine samples; Uosm, urine osmolality; Usg, urine specific gravity.

## Discussions

Various previous studies have mainly used urine colour charts for subjective qualitative analysis of urine colour. In the present study, an objective and accurate quantitative analysis of urine colour L*a*b* was performed using a benchtop spectrophotometer (Figure 1). The instrument has high precision and can identify colour difference values that are difficult for the human eye to recognise. The instrument uses the built-in pulsed xenon lamp to transmit the cuvette containing the urine sample, it uses the spectral sensor and microcomputer to analyse the colour and convert the colour space XYZ value to the L*a*b* value, respectively. Then, data are imported to the computer through the Spectra Magic NX software. To our knowledge, this is one of the first studies to analyse urine colour L*a*b* parameters amongst young athletes. Our subjects are all elite athletes from the Chinese national and provincial teams, with a large sample size covering different genders, regions and sports events.

Currently, there is no unified ‘gold standard’ for assessing hydration status (4). Of the studies on urine colour, the majority of studies used urine specific gravity and urine osmolality as diagnostic criteria for assessing hydration (6). For example, Armstrong et al.(3) defined urine specific gravity and urine osmolality as the criteria for assessing hydration. They found that the urine colour was highly correlated with urine specific gravity and urine osmolality by testing the spot urine of nine males during exercise and rehydration, and analysing the correlation between urine colour score and urine specific gravity, urine osmolality. It was confirmed that the urine colour chart could be used to assess the hydration of athletes, and urine colour was a valid index for assessing hydration. In our study, urine specific gravity and urine osmolarity were used as the diagnostic criteria of hydration for assessing the validity of urine colour L*a*b* parameters. In addition, our study found that there was a strong correlation between urine specific gravity and urine osmolality (r = 0.973, *p* < 0.01), and there was a univariate linear regression equation (Uosm = –35100 + 35100 Usg, R^2^ = 0.947). This suggests that urine specific gravity could be approximately converted to urine osmolality through a regression equation.

This study found that urine samples were assessed differently when urine specific gravity and urine osmolarity were used as the diagnostic criteria for hydration. Hew-Butler et al. (22) defined serum Na^+^ concentration >145 mmol/kg, urine specific gravity ≥1.020, and urine osmolarity ≥700 mmol/kg as the diagnostic criteria for dehydration. In addition, they detected the blood and urine indicators of 318 college athletes. The results for the number of people diagnosed with dehydration were not the same, which is similar to the results of this study. Therefore, false positive or false negative results will occur only with a single hydration evaluation standard. It is recommended to select various appropriate detection methods and analyse the results of multiple indicators when evaluating hydration amongst young athletes.

Through correlation analysis, we found that compared with L* and a* value, the b* value has the highest correlation coefficient with urine osmolarity and urine specific gravity; the validity of urine colour b* value in assessing hydration was high, the validity of urine colour L* value in assessing hydration was average, and urine colour a* value was not feasible for assessing hydration (Figure 3). Recently, Belasco et al. (12) collected a total of 151 urine samples under different stages of hydration from 28 healthy subjects (22 males and 6 females) aged 20–67 years and used a Hunter Lab Vista spectrophotometer to test their urine colour. The results showed that there was a significant linear relationship between urine colour b* value and urine osmolarity (*r* = 0.708, *p* < 0.05), which coincide with the results of this study; there was a moderate negative correlation between urine colour L* value and urine osmolarity (*r* = −0.567, *p* < 0.05), which was similar to the results of this study; there was also a low negative correlation between urine colour a* value and urine osmolarity (*r* = −0.375, *p* < 0.05), which was slightly different from the results of this study. Liu et al. (14) conducted a cross-sectional study on a total of 525 college students aged 17–23 (59 males, 466 females). They collected a total of 524 urine samples at different time points, imaging equipment was used to obtain urine images CIE L*a*b* and a Deere H-800 urine analyser was used to detect urine specific gravity. They found that urine specific gravity was positively correlated with b* value (*r* = 0.614, *p* < 0.01) and negatively correlated with L* value (*r* = –0.620, *p* < 0.01), which coincide to the results of this study. However, there was a moderate correlation between urine specific gravity and a* value (*r* = 0.664, *p* < 0.01), which was slightly different from the results of this study. Overall, quantitative analysis of urine colour L*a*b* was an effective method for assessing hydration amongst athletes. Compared with L* and a* values, the b* value had the highest correlation coefficient with urine osmolality and urine specific gravity. This suggests that the b* value had the highest validity compared with L* and a* values for assessing hydration in athletes. Differences in correlation coefficients in similar studies (11, 13, 14) might be related to factors such as detection instruments, cuvette light path, sediment deposits in urine samples, sample size and subjects.

The ROC curve further proved that the urine colour b* value could be used as an effective indicator for assessing different stages of hydration, and L* and a* values were not feasible to distinguish the hydration amongst athletes (Figures 4, 5 and Tables 2, 3). Zhang et al. (13) used urine osmolarity as a criterion for diagnosing hydration. After analysing a total of 413 spot urine samples of healthy college students, they found that urine colour b* value had good sensitivity and specificity for identifying hypohydration and hyperhydration, which was close to the results of this study. Currently, no studies have analysed the validity of urine colour L* and a* values for diagnosing different stages of hydration. Our study is the first to analyse the validity of L* and a * values for diagnosing different stages of hydration. It may be due to the validity of L* and a* values in diagnosing hydration is not that high, hence, it was not mentioned in the current study.

Recently, researchers (23, 24) developed a urine hydration system based on urine colour RGB. The system measures the urine colour through components such as colour sensors and urine test probes, infers the hydration state by analysing the B value of the urine colour RGB and uploads the test results to the mobile phone. This study found that there was a good fitting equation between the b* value and the urine concentration. We developed a urine hydration system based on the urine colour L*a*b* based on the regression equation (*b* = −1220 + 1220Usg, *R*^2^ = 0.657) and hydration level. We set thresholds for diagnosing different stages of hydration based on the b* value. The system used a colour sensor or digital camera to identify the L*a*b* value of the urine sample, and analysed the b* value and hydration level through the software to assess the hydration amongst athletes. In addition, we also developed a mobile application based on the b* value, which could roughly assess the hydration of the user by simply taking a picture of the urine sample and analysing the urine colour b* value. Considering the limited length of our paper, we could not give a detailed description. In the future, we can also develop more hydration detection devices based on L*a*b *thresholds or other colour space models for different groups of people.

Limitations of the current study include that the indicators selected were limited. In the future, we can add more indicators, such as other colour space models, urine and blood indicators. It was undeniable that confounding factors including urine test time, subject age, gender and training might affect the results. In addition, our study only focused on young athletes, and further studies can be conducted in different age groups in the future.

## Conclusion

This study demonstrated that the validity of urine colour b* value for assessing hydration in athletes was high, however, the validity of urine colour L* and a* value was low based on the correlation and ROC curve analysis results. In the future, the b* value can be used to diagnose hydration amongst athletes, and we can also use it to develop hydration detection equipment to serve athletes and even the general population.

## Data availability statement

The raw data supporting the conclusions of this article will be made available by the authors, without undue reservation.

## Ethics statement

The studies involving human participants were reviewed and approved by Human Experiment Ethics Review Committee of the China Institute of Sport Science (Approval Number: 20220113). The patients/participants provided their written informed consent to participate in this study.

## Author contributions

YF, GF, FQ, and JZ contributed to the conception and design of the study. SC, CQ, and XG assisted in completing the experiment. YF performed the statistical analysis and wrote the first draft of the manuscript. GF, XG, DG, and JZ revise the manuscript. All authors contributed to manuscript revision, read, and approved the submitted version.

## Abbreviations

Uosm: urine osmolality; Usg, urine specific gravity.
