# Supplementary figures and images for: Validation of urine colour L*a*b* for assessing hydration amongst athletes
Source: Front Nutr. 2022 Aug 10;9:997189. doi: 10.3389/fnut.2022.997189 (PMC9399725; doi:10.3389/fnut.2022.997189)

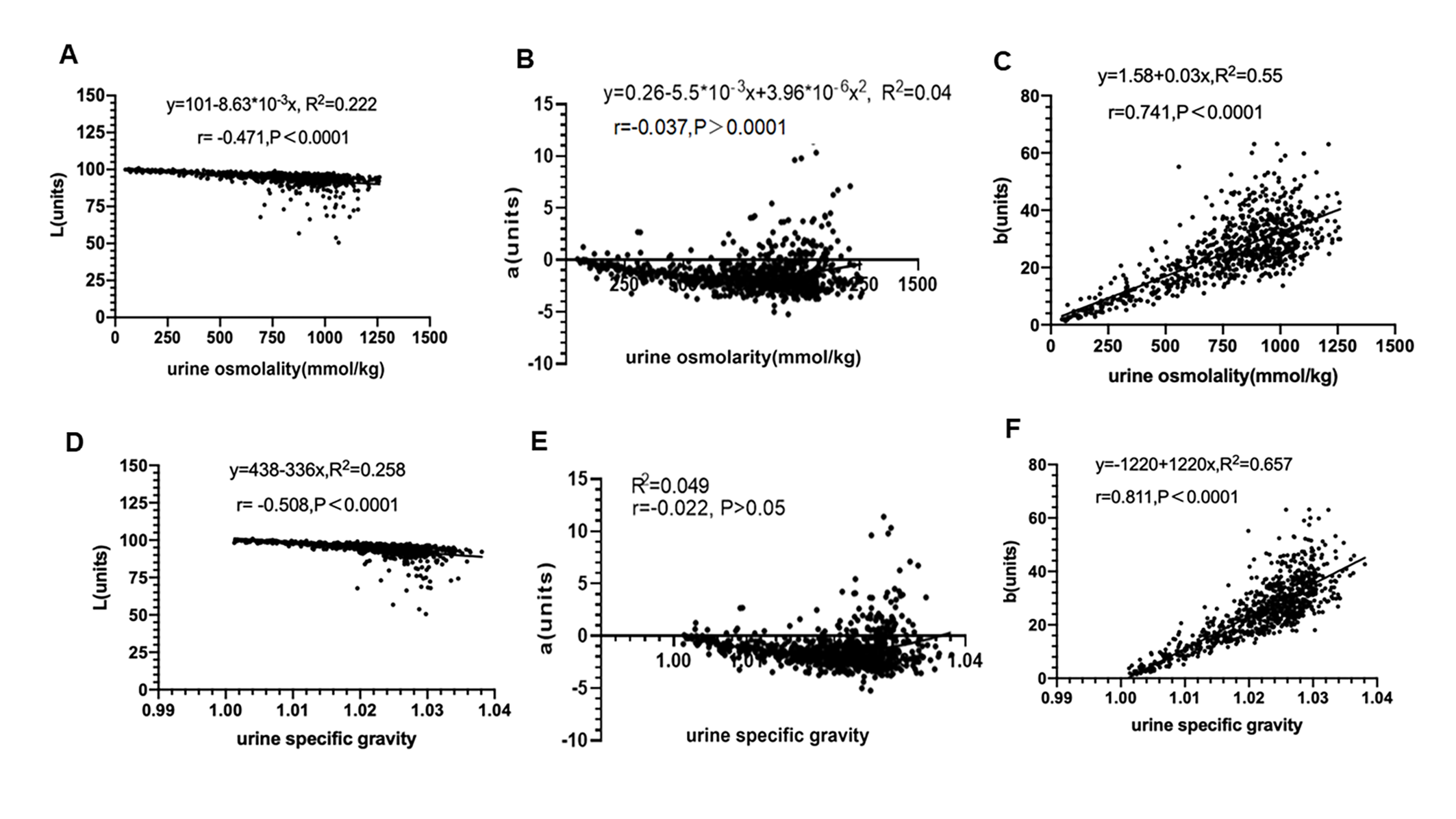

Supplement: Supplementary file 1 [file Data_Sheet_1.ZIP › Supplementary Material Presentation/FIGURE 3.TIF]

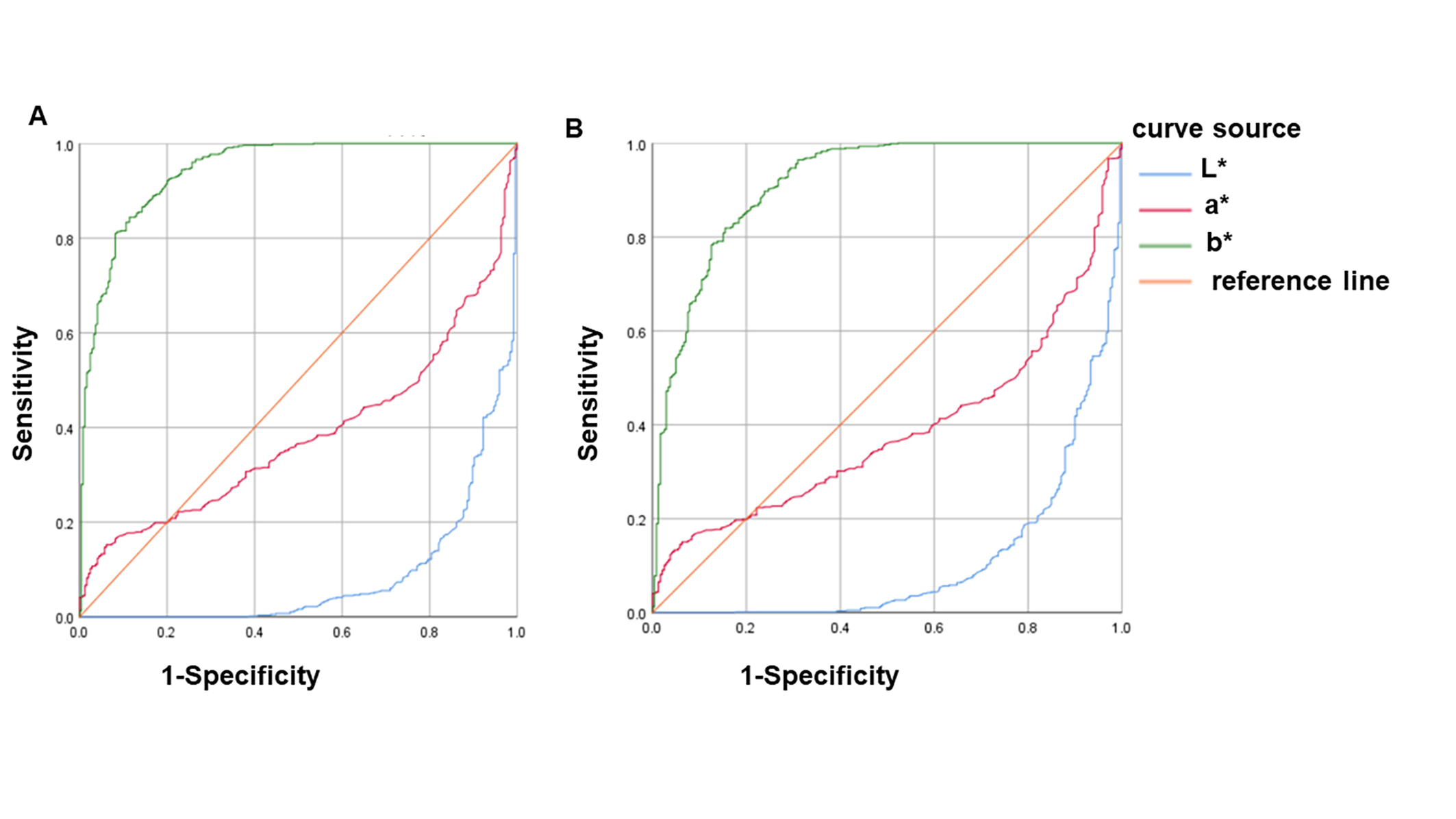

Supplement: Supplementary file 1 [file Data_Sheet_1.ZIP › Supplementary Material Presentation/FIGURE 4.TIF]

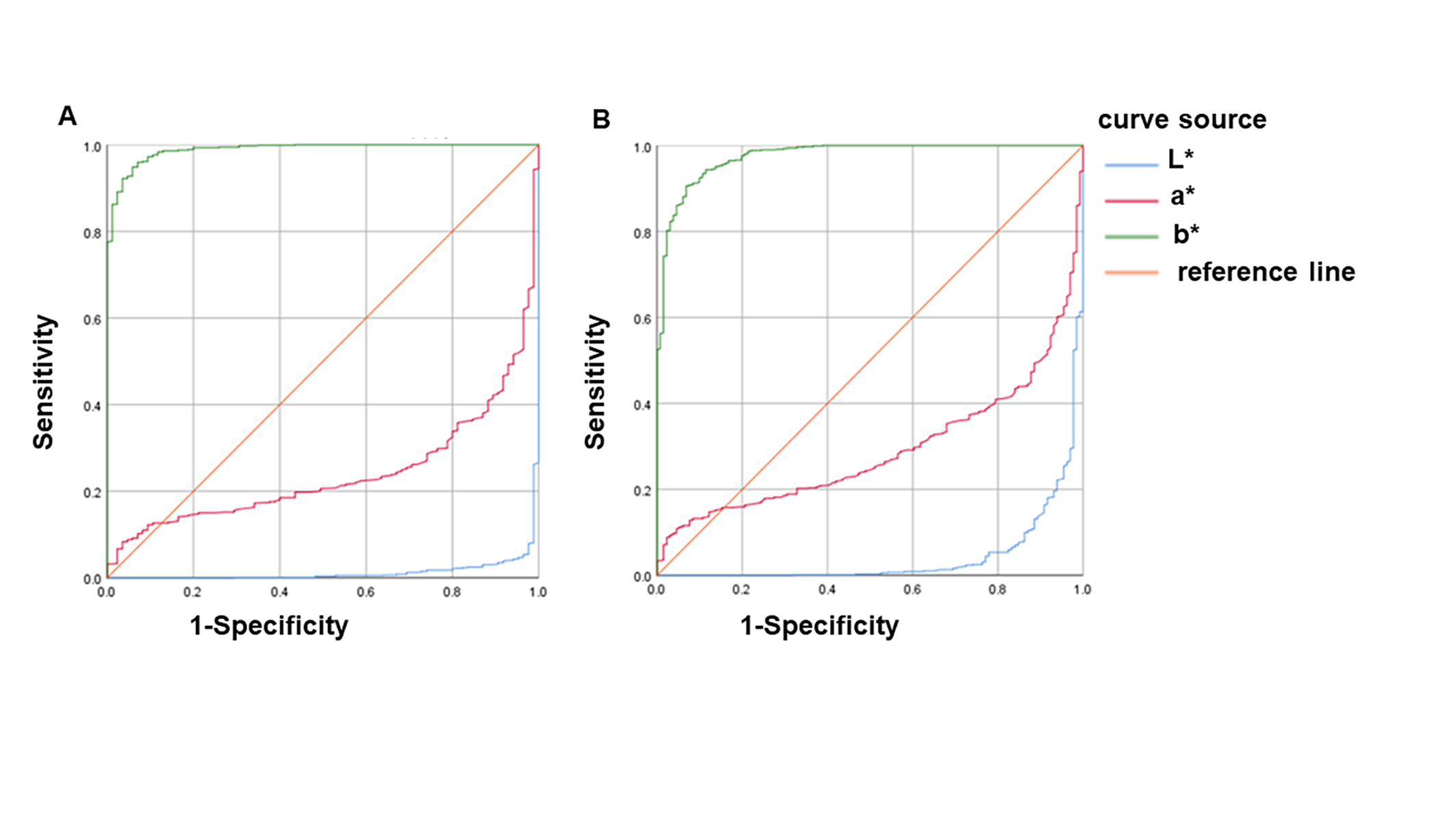

Supplement: Supplementary file 1 [file Data_Sheet_1.ZIP › Supplementary Material Presentation/FIGURE 5.TIF]

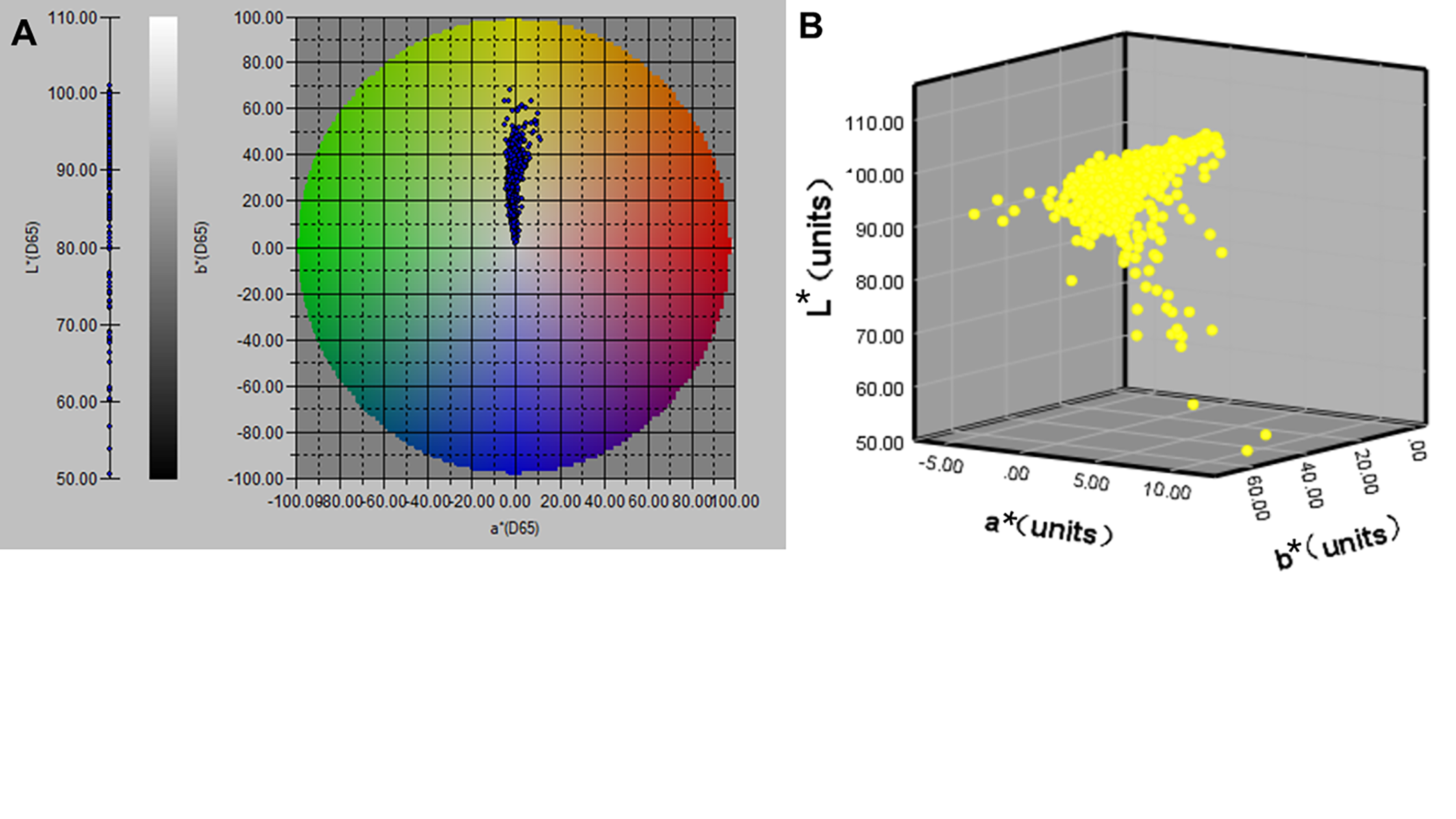

Supplement: Supplementary file 1 [file Data_Sheet_1.ZIP › Supplementary Material Presentation/Figure 1.tif]

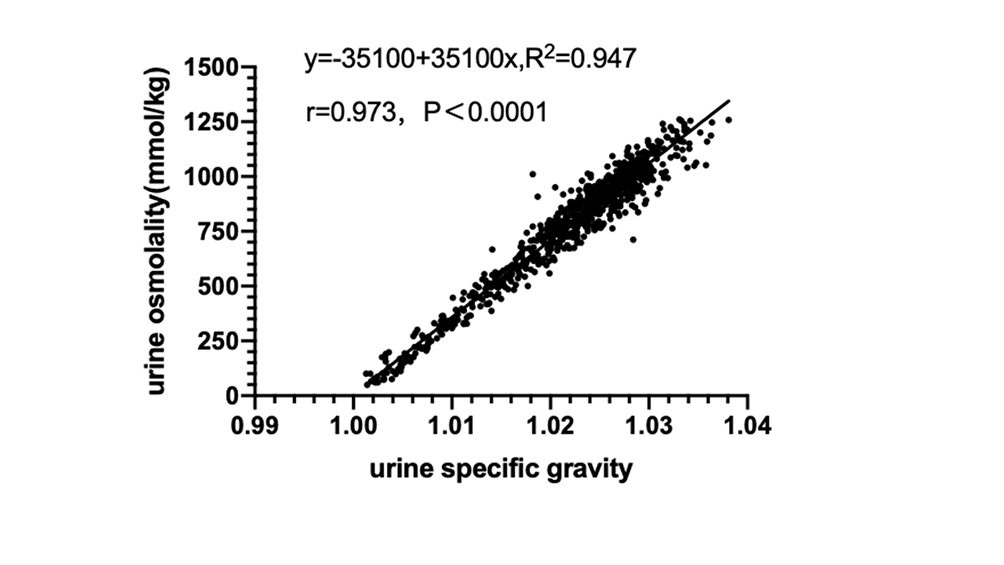

Supplement: Supplementary file 1 [file Data_Sheet_1.ZIP › Supplementary Material Presentation/Figure 2.tif]
